# A protocol for microRNA extraction from gastrointestinal digesta

**DOI:** 10.1016/j.fochms.2025.100245

**Published:** 2025-02-11

**Authors:** Miguel Cifuentes Acebal, Yvan Devaux, Torsten Bohn

**Affiliations:** a Nutrition and Health Research Group, Department of Precision Health, Luxembourg Institute of Health, Strassen, Luxembourg; b Doctoral School in Science and Engineering, University of Luxembourg, 2, Avenue de l'Université, 4365 Esch-sur-Alzette, Luxembourg; c Cardiovascular Research Unit, Department of Precision Health, Luxembourg Institute of Health, Strassen, Luxembourg

**Keywords:** In vitro digestion, INFOGEST, Digestive enzymes, Intestine, Nucleotides, XenomiR

## Abstract

MicroRNAs (miRNAs) are non-coding RNAs that influence gene-expression via post-transcriptional regulation of target protein-coding RNAs. With literature reports indicating survival of diet-derived miRNAs following their ingestion, it is important to study their stability and concentration during gastrointestinal digestion. The unique combination of chemicals and elevated RNAse content present in the gastrointestinal matrix may be a limiting factor for studying diet-derived miRNAs. First, chemical cross-reactivity with matrix constituents (e.g. bile salts) may interfere with the salt bridge interactions typically present during RNA extraction, reducing the efficiency of the column. Second, high RNAse content may not be fully inhibited during extraction and could continue degrading the miRNAs, as is observed for other tissues with high RNAse content. These combined issues may result in a reduced efficiency in yield and purity of RNA extracts, further limiting the study of diet-derived miRNAs (i.e. in downstream metabolism). In the present manuscript, we display a method based on silica column purification to extract and quantify diet-derived miRNAs from the bioaccessible phase of the gastrointestinal digesta. The proposed protocol provides a simple, quick (less than 2 h), reliable, and systematic method for miRNA purification from gastrointestinal digesta. The optimization showcased that the challenges caused by high RNAse activity, plant bioactive substances and bile-salt content within the gastrointestinal digesta have been overcome and the study of the miRNA fraction in a body fluid so far neglected is now available to researchers, allowing the use of miRNA as biomarkers of intake and potentially biomarkers of biological changes.

## Highlights

• Gastrointestinal matrix reduced efficiency for miRNA extraction.
• Main causes for decreased yield were high RNAse content and cross-reactivity.
• Novel approach overcome previously observed limitations in RNA extraction.
• Protocol allowed study of diet-derived miRNAs.

## 1 Introduction

MicroRNAs (miRNAs) are single stranded non-coding RNAs containing 21–24 nucleotides that are detectable in most multicellular eukaryotic organisms and able to regulate genes at the post-transcriptional level (Cortez et al., 2011; Shabalina and Koonin, 2008). They are detectable in human body fluids such as blood, saliva, and urine, and their presence or absence has been associated with disease states and even have the potential to be used as disease biomarkers (Caporali et al., 2024). Thus, the characterization of miRNA profiles has been a key strategy for gaining insights into many diseases, including cancer and cardiometabolic diseases (Goretti et al., 2014; Li et al., 2023). In this context, the presence of exogenous miRNAs circulating in human blood was first detected more than a decade ago (Zhang et al., 2012). At first, these miRNAs were reported to be of plant origin (Liang et al., 2014; Zhang et al., 2012), although exogenous miRNAs of animal origin were later reported as well (Zhang et al., 2019). These findings gave way to the “dietary xenomiR hypothesis”, proposed soon after the first reports were published, which suggested that miRNAs derived from food items can survive gastrointestinal digestion and reach human body compartments (Witwer, 2012).

The ubiquitous detection of miRNAs through different phyla translates to these being present in most food items of both animal and plant origin such as rice, cabbage, or milk (Jia et al., 2021). Nonetheless, miRNA stability in the food matrix or their state is not fully understood and little data is available. Especially if the environmental widespread of RNAses is considered (also in the gastrointestinal tract), as these will quickly degrade miRNAs (Houseley and Tollervey, 2009). However, miRNAs may be detected in extracellular vesicles such as exosomes (Mohr and Mott, 2015; Vishnoi and Rani, 2017). Exosomes are thought to play an important role in improving diet-derived miRNA stability (Del Pozo-Acebo et al., 2021; Jia et al., 2021). In any case, uptake by humans would require miRNAs to be released from the food matrix, followed by transport and secretion at the basolateral site of the enterocytes. Data from endogenous miRNAs in other body compartments, where absorption takes place via endocytosis, suggests that a similar process may occur in the intestinal epithelia (Zhang et al., 2010). Nonetheless, the specific uptake mechanism of diet-derived miRNAs in enterocytes has not been studied to date, and absorption through endocytosis remains only a hypothesis (Del Pozo-Acebo et al., 2021).

With a growing body of literature demonstrating the presence of diet-derived miRNAs in human and animal plasma, several authors have started to study the plausibility of the dietary xenomiR hypothesis. This has included the in vitro study of miRNA survival to mastication and salivary digestion (Qin et al., 2022), as well as the gastric phase (Liao et al., 2017; Philip et al., 2015), and even the intestinal phase in mice (Liang et al., 2014) and excretion in human feces (Diez-Sainz et al., 2024). Nevertheless, and although miRNA survival has to some extent been demonstrated in all of these scenarios, a standardized method for miRNA isolation and purification from gastrointestinal digesta (GID), is lacking. Such a method would allow for a comprehensive and reproducible study of diet-derived miRNAs, and to test their stability during food processing in the gastrointestinal tract.

Achieving an efficient isolation of miRNAs derived from food matrices poses major challenges. First, the complexity of the GID matrix, which is rich in bile acids, salts, amino acids/peptides, phospholipids and undigested proteins, may interact with and disrupt the extraction of RNA. This has been observed for other complex matrices such as liver, where glycogen may co-precipitate with RNA, lowering the yield and purity (Sharma et al., 2019). Second, the high concentration of minerals and digested plant matrix materials, including secondary plant bioactives, among others, could further hinder the purification process, either during RNA precipitation or column cleanup. Finally, RNAses, which are common within the GID (both endogenous and exogenous RNAses), may compromise the integrity of RNA during the purification, as previously reported in other tissues with high RNAse concentration (Jun et al., 2018).

Therefore, the aim of the present article is to present a simple, rapid, and reproducible protocol for miRNA extraction from the GID. For this purpose, the static INFOGEST model was used (Brodkorb et al., 2019). This model, similar to other static in vitro digestion models, is frequently employed by researchers to digest complex food matrices. Although the present protocol has been developed with a special focus on miRNAs purification, it also extracts total RNA. It has been optimized to be fully compatible with widely applied downstream applications, such as reverse transcription followed by quantitative PCR (RT-qPCR). The protocol requires an input volume of 600 μl of GID and is achieved in less than 2 h.

## 2 Materials and methods

### 2.1 Chemicals and consumables

Unless otherwise stated, the consumables and chemicals used were of molecular biology grade or comparable. MiRNeasy Serum/Plasma Advanced kit (217184), miRCURY LNA SYBR ® Green PCR Kit (339347), and miRCURY LNA RT Kit (339340) were purchased from Qiagen (Hilden, Germany). RNAsecure ™ Reagent, and miRVana ™ miRNA mimic cel-mir-39 were obtained from Thermo Fisher scientific (Waltham, MA, US).

Chemicals were purchased from Sigma Aldrich (St. Louis, MO, US), including KCl KH_2_PO_4_, NaHCO_3_, NaCl, MgCl_2_(H_2_O)_6_, (NH_4_)_2_CO_3_, CaCl_2_(H_2_O)_2_, porcine pepsin (powder, 250 U/mg, Cat. P7000), porcine pancreatin (activity equivalent to 4× USP specifications, Cat B1750), porcine bile (B8631), as well as chloroform, isopropanol 99.5 %, and ethanol 100 %.

### 2.2 Sample description and pre-processing

Cauliflower (*Brassica oleracea* var. *botrytis*) was chosen due to previous reports of detected miRNA (*B. oleracea*) found in mice plasma (Liang et al., 2014). Frozen pre-cut cauliflower was bought at a local supermarket (Delhaize, Strassen, Luxembourg). Immediately upon arrival to the laboratory, it was homogenized for a few seconds using a commercial kitchen grinder (La Moulinette DPA141, Moulinex, Alençon, France) to obtain a smaller and homogenous particle size and to simulate mastication. For one set of digestions, the plant material was then separated into aliquots of 4 g each and stored at −80 °C until in vitro gastrointestinal digestion was conducted.

### 2.3 Gastrointestinal digestion

In vitro gastrointestinal digestion was performed following the static INFOGEST 2.0 model, which has been described elsewhere (Brodkorb et al., 2019), with minor modifications. The oral phase was omitted due to preceding homogenization and the matrix being low in carbohydrates. In short, homogenized, aliquoted samples (4 g cauliflower) were first treated with a simulated gastric fluid (SGF) (KCl 6.9 mM, KH_2_PO_4_ 0.9 mM, NaHCO_3_ 25 mM, NaCl 47.2 mM, MgCl_2_(H2O)_6_ 0.1 mM, (NH_4_)_2_CO_3_ 0.5 mM, CaCl_2_(H_2_O)_2_ 0.075 mM, pepsin 2000 U/ml) at pH 3,adjusted with 0.1 mM HCl. The food matrices were incubated at 37 °C in SGF for 2 h in a shaking bath at 100 rpm.

Following gastric digestion, a simulated intestinal fluid (SIF) (KCl 6.8 mM, KH_2_PO_4_ 0.8 mM, NaHCO_3_ 85 mM, NaCl 38.4 mM, MgCl_2_(H_2_O)_6_ 0.33 mM, CaCl_2_(H_2_O)_2_ 0.3 mM, pancreatin 200 U/ml, bile extract 6.8 mg/ml) was added to the SGF. pH was then adjusted to 7 with 0.1 mM NaOH. This incubation was done following the same conditions (time, rpm) as before. The final volume of the GID was adjusted with water to 26 ml. After incubation, the samples were centrifuged at 4 °C and 3200 *g* for 1 h to precipitate any large particles present.

Finally, the aqueous phase of each sample was collected (approx. 9 ml) and filtered through a 0.2 μm nylon filter to produce the bioaccessible phase. To reduce variability between samples caused by the digestion, assure same starting levels of miRNA and to study variability related to the employed protocol, bioaccessible phases were pooled together prior to filtration. As a result, a pooled sample was produced. This phase was aliquoted into 1.8 ml samples, collected in 2 ml PCR clean tubes (Eppendorf, Hamburg, Germany) and frozen at −80 °C.

### 2.4 Extraction procedures for RNA

#### 2.4.1 Method retained

RNAsecure™ reagent was used for RNA stabilization and protein degradation prior to any extraction began. Incubation with RNAsecure was performed for 10 min in a preheated Eppendorf ThermoMixer F2.0 at 60 °C to promote denaturation of RNases and proteins. The RNA purification was conducted using the miRNeasy Serum/Plasma Advanced kit as a starting point. The kit uses a combination of lysis (Buffer RPL) and precipitation (Buffer RPP) buffers containing guanidine thiocyanate and detergents to lysate cells and other structures containing miRNAs (i.e. exosomes) while removing proteins and other contaminants (i.e. RNAses). Later steps include silica column binding (RNeasy UCP MinElute Spin Column), washing (Buffer RWT, Buffer RPE, 80 % ethanol), and final elution in 20 μl of RNAse free water. The eluate's total RNA concentration was quantified by UV–Vis spectrophotometry (Nanodrop, Thermo Fisher Scientific, Waltham, MA, US) and a Qubit microRNA assay kit (Invitrogen, Waltham, MA, US) for the total miRNA fraction. In addition, 3.5 μl of spike-in (cel-mir-39, 2*10^10^ copies/μl) were added at the beginning of the extraction to control for the purification efficiency. The centrifugation steps were performed with a fixed-angle Eppendorf micro-centrifuge capable of reaching 16,000 x*g*. Due to reports from the manufacturer that incubation of the samples with DNase I (Qiagen, Hilden, Germany) may cause loss of miRNAs, we evaluated the yield in both scenarios (with and without DNase treatment).

#### 2.4.2 Methods used for comparison

Additionally, the new protocol was benchmarked against common techniques used for miRNA extraction, including phenol-chloroform precipitation with resuspension in water (Chomczynski and Sacchi, 2006), and phenol chloroform combined with spin column purification (Karp et al., 1998). The phenol-chloroform extraction followed by a column cleanup (Method 1) was carried out using the miRNeasy mini kit (Qiagen, Hilden, Germany) following manufacturer's guidelines, with minor modifications. In short, 750 μl of Qiazol (included in the kit) were added to 250 μl of GID. The spike-in cel-mir-39 was added at this point and incubated for 5 min at room temperature. Next, 200 μl of chloroform were added to the mixture, followed by shaking, incubation (3 min) and centrifugation at 4 °C for 15 min (12,000 x g). The supernatant was then transferred to a Min Elute Spin Column (Qiagen) and a sequence of binding buffers and wash buffers was applied according to the manufacturer's protocol. Final elution was achieved into a volume of 30 μl RNase free water.

For the phenol-chloroform extraction without spin column (Method 2), we followed the manufacturer's guidelines, again with minor modifications: Cold (4 °C, 750 μl) TRIzol LS ™ (Invitrogen, Carlsbad, CA, US) reagent was added to 250 μl of GID. The mixture was then gently mixed and incubated at room temperature for 15 min. Afterwards, 200 μl of chloroform were added to the sample, followed by gently mixing and 5 min incubation at room temperature. The organic phase was precipitated in a centrifugation step (12,000 x*g*, 4 °C, 15 min), and the aqueous phase was then transferred to a new tube. To this phase, 500 μl of isopropanol 99.5 % were added and the mix was incubated for 10 min at room temperature. After incubation, another centrifugation (12,000 x*g*, 4 °C, 10 min) took place. The supernatant was discarded, and the pellet resuspended in 1 ml 75 % ethanol. After mixing, another centrifugation followed (7500 x*g*, 4 °C, 5 min). Next, the supernatant was discarded, and the pellet was air dried for 10 min at room temperature. Finally, it was resuspended in 20 μl of RNAse free water. For both methods (i.e. 1 and 2), the extractions were performed both with 250 μl of pure GID and with GID that had previously been incubated at 60 °C for 10 min with RNA secure (240 μl GID +10 μl RNA secure).

#### 2.4.3 Sample selection

The 1.8 ml aliquots of GID produced earlier contained sufficient material to perform four RNA extractions. Thus, each aliquot was used to produce RNA extracts derived from methods 1 and 2, as well as our new method and its counterpart without RNAsecure.

### 2.5 Reverse transcription and qPCR

Reverse transcription was carried out using miRCURY LNA RT Kit, following the manufacturer's recommendations, loading a sample volume of 6.5 μl and a total reaction volume of 10 μl. Reverse transcription was performed in a C1000 Touch Thermal Cycler machine (Bio-Rad, California, US). Two reactions were performed as RT negative controls, one sample with no RT enzyme supplied, and one sample with RT enzyme but no RNA template.

The resulting cDNA was diluted 60 times in RNAse free water and PCR was conducted using the miRCURY LNA miRNA PCR assay. A sample with no cDNA was also added to the PCR plates as a negative control. An exogenous miRNA (UniSp6, supplied with miRCURY LNA RT kit) was spiked-in and used as control of RT, following the manufacturer's guidelines (0.5 μl per sample, supplied directly to the RT master mix). A miRNA endogenous to cauliflower (bol-mir-172a) was used to assess miRNA extraction from the GID. PCR primers were ordered from Qiagen (miRCURY LNA miRNA PCR assay). PCR was conducted in a Bio-Rad CFX96 Touch Real-Time PCR Detection System. For miRNAs in the GID (i.e. bol-mir-172a), cel-mir-39 was used as an inter-sample calibrator, to account for technical variability (∆Cq = Cq GIDbol-mir-172a- Cq cel-mir-39). The calculation of the number of copies of cel-mir-39 was carried out following a standard curve created by a serial dilution of said miRNA, starting at 10^8^ copies until 10^1^copy. From this curve, the following equation was extracted: Number of copies =10^ ((Cq-39.497)/−3.449). Every sample was loaded two times into the PCR plate for each primer, to produce a technical replicate of the PCR. The data was only considered valid if the deviation between technical PCR replicates was below 0.3 cycles. All samples were adjusted to this criteria. The final Cq value for each sample was calculated as the average of the technical replicates.

### 2.6 Statistics and software

RStudio was used to conduct statistical testing (R version 4.3.2). The different groups were analyzed using Wilcoxon signed-rank test, with a predefined alpha value of 0.05 (2-sided). When conducting multiple testing, *P*-values were corrected to adjust for the false discovery rate following the Benjamini-Hochberg principle. Cohen's d effect size calculations were performed on samples that were statistically significant. Corrected *p*-values are reported both in the text and in the figures. Plotting was done using the ggplot2 package and Inkscape.

## 3 Results

### 3.1 MicroRNA extraction protocol from GID

**Fig. 1:**
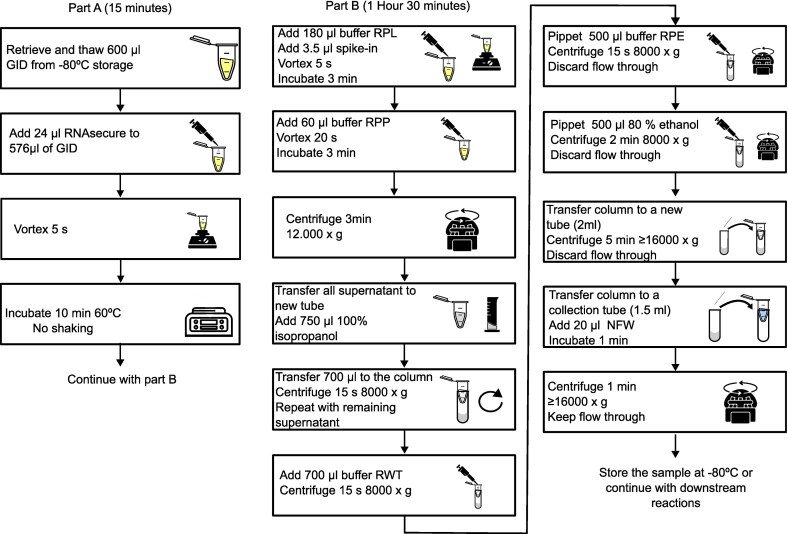
Workflow to purify miRNAs from gastro intestinal digesta in less than two hours. The protocol is divided into two parts; the approximate length of each part is indicated on top. A break can be introduced between Part A and Part B. Unless otherwise specified, all steps are conducted at room temperature.

The protocol consists of two main parts. It allows for a maximum tested input volume of 600 μl of GID and requires a total processing time below 2 h (Fig. 1). The initial phase of the protocol (Fig. 1A) aims to reduce the degradation of the miRNAs present in the digesta due to RNAses, and to remove cross-reactivity with other proteins in the GID. The second part of the protocol extracts and concentrates miRNAs from the GID and is optimized for use with downstream applications (i.e. RT-qPCR).

**Fig. 2:**
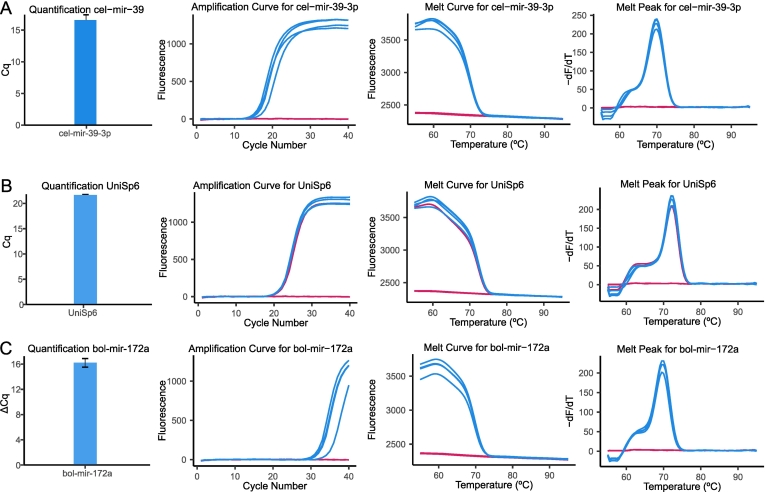
RT-qPCR data relative to different miRNAs. In blue data from the samples, in red negative controls. A: Data regarding the spike-in cel-mir-39-3p, used as a control for RNA extraction. From left to right absolute quantification (in log scale), amplification curve, melt curve, melt peak. B: Data regarding the spike-in UniSp6, used as RT control. From left to right quantification (as cycle threshold) of the spike in, amplification curve, melt curve, melt peak. C: Data corresponding to bol-mir-172a, a miRNA present in the digested matrix. From left to right: Relative cycle quantification (∆Cq) with cel-mir-39 as a calibrator, amplification curve, melt curve, melt peak. (For interpretation of the references to colour in this figure legend, the reader is referred to the web version of this article.)

Reduction of RNase activity and other possible contaminants is achieved by the addition of 24 μl of RNAsecure™ (Invitrogen, Vilnius, Lithuania) to 576 μl of sample (X 24 dilution of stock solution). The mixture of RNAsecure + GID is then incubated for 10 min at 60 °C to promote inactivation of RNAses and reduction of proteins present in the GID through thiol group interactions. This is a critical step in the method. Not adding RNAsecure at the start of the protocol leads to a decreased reproducibility and yield in the extraction/quantification of miRNAs (Fig. 2). Samples to which RNA secure was not added showed a great variability in the recovery of the spike-in cel-mir-39 (Cq = 20.6; SD = 15.7), while samples treated with RNAsecure tended to group together with lower variability (Cq = 16.6 SD = 0.8) (*P* = 0.04) (Fig. 2A).The same statement is also true for UniSp6 (*P* = 0.029)(Fig. 2B) and bol-mir-172a (*P* = 0.03)(Fig. 2C). In case of the bol-mir-172a when RNAsecure was not added, the miRNA could not be detected in any of the samples. Contrarily, if incubation with RNA secure took place, bol-mir-172a was detected across all samples with a low standard deviation (Cq = 32.77 SD = 1.49) (Fig. 2 C).

Within the second part of the protocol, RNA extraction is achieved (Fig. 1B). To this end, we utilized a commercial kit developed for the extraction of miRNAs from blood and plasma samples (miRNeasy Serum/Plasma Advanced kit). First, 180 μl of lysis buffer RPL are added to the 600 μl derived from previous steps. At this point, the spike-in control is also added, in this case 3.5 μl of cel-mir-39. The sample is then mixed with a vortex for 5 s and incubated at room temperature for 3 min. Later, 60 μl of precipitation buffer RPP are added to the sample. The mix is vortexed for 20 s and incubated at room temperature once more.

It must be noted that a white precipitate is formed after addition of Buffer RPP (Supplementary Document I, Part B, Step 5). This precipitate is eliminated by centrifugation (12,000 x*g*, 3 min), after which the supernatant is retained. Residual material is also found at the tube walls and at a thin low-density layer on top of the aqueous phase. The supernatant (approx. 750 μl) is transferred to a new micro centrifuge tube. It is important to avoid touching the pellet and tube walls to avoid aspiration of precipitate. Furthermore, pipetting directly from the surface should not be done, to avoid carryover of the low-density residue. Carryover of any of the residues will affect the yield and purity. Afterwards, add 1 volume (Usually 750 μl) of 99.5 % isopropanol to the supernatant and mix by vortexing. From this mixture, transfer a maximum of 700 μl to an RNeasy UCP minElute column. Then, centrifuge for 15 s at 8000 x*g*. Discard the flow through and repeat the previous step until the entire mixture has been centrifuged.

In the following step, DNAse I digestion is conducted: add 350 μl of wash buffer RWT (diluted with 45 ml of 99.5 % isopropanol instead of 30 ml 100 % ethanol) and centrifuge with the same parameters as before (8000 x g, 15 s). Then, discard the flow-through and incubate for 15 min at room temperature with 10 μl of DNase I + 70 μl of buffer RDD. After the incubation period add 500 μl of buffer RWT (diluted with isopropanol) to the column and centrifuge with the same parameters. Save the flow-through for the next step. Place the column in a new collection tube and then add the flow-through to it and centrifuge once more.

If no DNase I digestion is performed, continue from here. Add 700 μl of wash buffer RWT (prepared with 30 ml of 100 % ethanol) and centrifuge (8000 x*g*, 15 s). Discard the flow-through. Add 500 μl of wash buffer RPE, centrifuge (8000 x*g*, 15 s) and discard the flow-through. Continue washing with 500 μl of 80 % ethanol. Increase centrifugation time to 2 min, keep the same speed (8000 x g) and discard the flow-through. Change the collection tube. Centrifuge at 16000 x*g* and 5 min to fully dry the membrane. Discard the collection tube and flow-through. Place the column in a 1.5 ml collection tube. Add 20 μl of RNAse-free water directly to the membrane and incubate 1 min at room temperature. Finally, centrifuge at 16000 x*g* for 1 min and retain the eluate, as it contains the RNA. A step by step workflow of the protocol is available in the supplementary document I.

The eluate obtained at the end of the protocol was later analyzed using RT-qPCR. A preliminary analysis of the total RNA and miRNA concentration was carried out with UV–Vis (Nanodrop) and Fluorimetry (Qubit) respectively. Neither Nanodrop nor Qubit, could detect any concentration of RNA as it was below both instruments' detection limit. Thus, this data is not reported here as it had no effect on RNA quantification. Nonetheless, the absorption spectra of the nanodrop indicated significant contamination at 230 nm and 280 nm from samples extracted with all the methods listed, except for method 1, where the eluate showed an absorbance of 0 throughout the entire measured spectrum.

### 3.2 PCR quantification

**Fig. 3:**
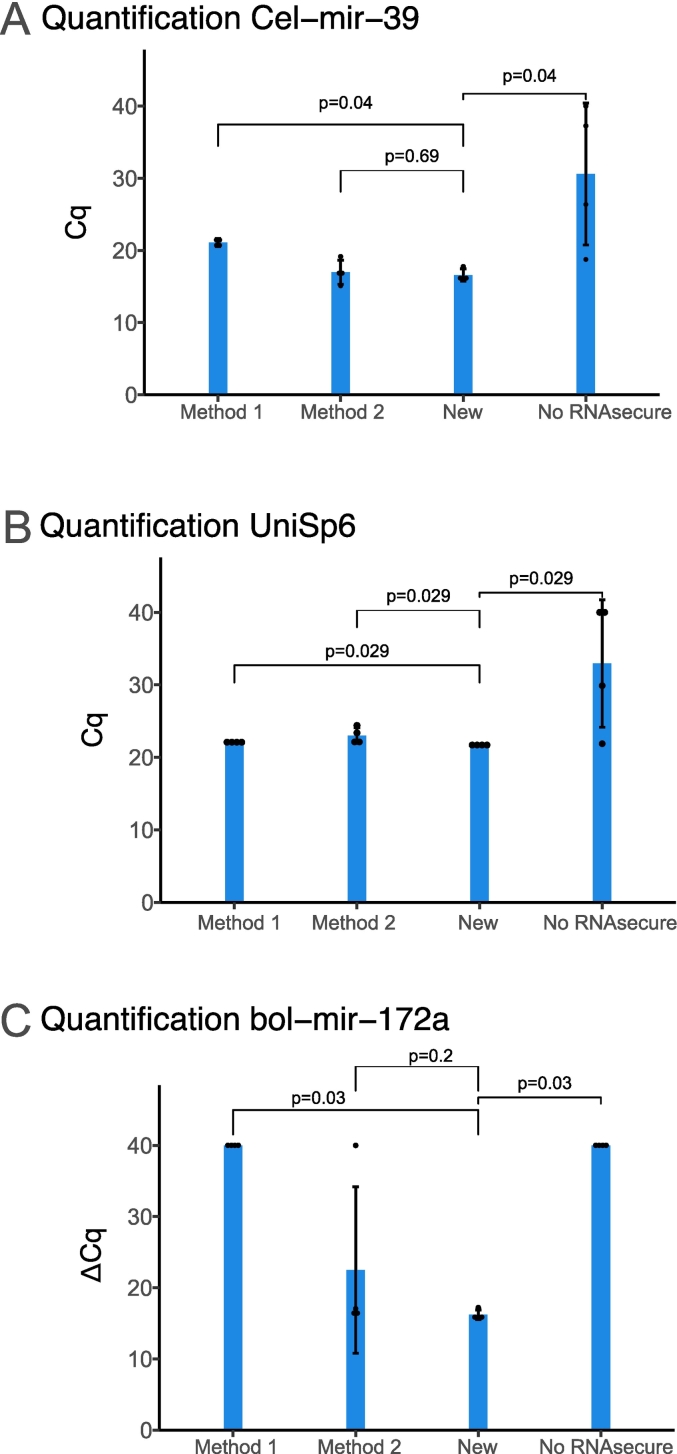
RT-qPCR data comparing four methods for RNA extraction from gastro-intestinal digesta (*N* = 4). Method 1: Samples where the miRNeasy mini kit extraction protocol was followed; Method 2: Samples extracted using TRIzol LS; New: Samples extracted with a novel extraction protocol defined in the manuscript; No RNA secure: Samples extracted in the same manner as New, but lacking the key step of RNA secure incubation. Horizontal lines indicate statistical significance. Error bars display standard deviation. A: Results for spike-in cel-mir-39. B: Results for spike-in UniSp6. C: Results for bol-mir-172a, shown as ∆Cq, using cel-mir-39 for normalization.

The analysis of the RNA extract was done by RT-qPCR for different miRNAs. First, the absolute quantification of the spike-in cel-mir-39 was performed to study RNA purification success. The spike-in was detected at Ct ≈ 16 (N° Copies = 4.37 * 10^6^ ± 2.26 * 10^6^) (Fig. 3A). Moreover, the amplification curve followed a normal path, indicating a successful reaction with a good amplification efficiency and absence of primer-dimer formation. Furthermore, the melt peak and melt curve confirmed these observations by following the expected behaviors for the target and being consistent across replicates. These signals are indicative of a successful and specific reaction (Fig. 2A). Meanwhile, the negative controls failed to produce a signal. Altogether, these results indicate a successful RNA purification from the sample. No significant change in concentration of the spike-in could be observed between samples processed with and without DNase I (Supplementary Fig. I, A).

The spike-in UniSp6, which was used as RT control and added to the RNA purified extract prior to RT, was detected uniformly across all samples (Ct = 21.65 ± 0.09) (Fig. 2B), indicative of a successful RT and lack of reaction inhibition. Furthermore, the amplification curve, melt peak, and melt curve for UniSp6 did not show abnormal behaviors (Fig. 2B). The two negative controls (RT enzyme free control and cDNA free control) failed to produce a signal. The third control (RNA free template) showed a normal positive signal, as this sample had been spiked with UniSp6 as part of the master mix.

Finally, plant-derived bol-mir-172a was analyzed. Equally to what cel-mir-39 quantification had indicated, DNase I treatment did not show a significant effect on yield (*P* = 0.34), although there was a trend for decreased reproducibility when compared to standard treatment, as shown by the increase in standard deviation (SD = 0.95 vs. 0.67, respectively), (Supplementary Fig. I, B). Even if there was no statistical difference, we do not recommend DNase I treatment when working with GID, especially if miRNAs of interest are weakly expressed. Therefore, only data from samples not treated with DNase I is reported here. All samples were within the detection limit of 35 cycles. Furthermore, after normalization, the samples could be detected in a replicable manner (Fig. 2C). Also for this spike, the QC measurements (melt peak, amplification curves, and melt curves) and negative controls behaved as expected.

### 3.3 Benchmarking of the protocol

In order to obtain a better idea on the performance of the method, we decided to compare it to other common techniques employed in total and miRNA extraction. Those methods suffered from various limitations when tested. First, method 1 had a lower yield of both spike-in controls cel-mir-39 (*P* = 0.04) and UniSp6 (*p* = 0.029) and bol-mir-172a (*p* = 0.03) than the optimized protocol (Fig. 3). Furthermore, a large effect size reinforcing these findings both for cel-mir-39 (d = 6.3) and bol-mir-172 (d = 6.8) has been calculated. Additional RT-qPCR data to the product of this method (Supplementary fig. II) indicates that both the qPCR and RT have been conducted successfully. Nonetheless, it was highlighted that bol-mir-172 (supplementary fig. II, C) had a lower reproducibility than the spike-ins or than samples processed with the new protocol. Taken together, the data indicates that method 1 is not as robust in extracting miRNAs that are weakly expressed within the GID compared to the method we have presented. We could not detect any significant differences between samples treated with or without RNAsecure if following this method.

Regarding method 2, no statistical difference regarding the detection of cel-mir-39 could be detected (*p* = 0.69) compared to the new method (Fig. 3A). In contrast, a significant difference could be observed between method 2 in the RT spike-in and the new method (*p* = 0.029) (Fig. 3B). Finally, the statistical significance disappeared when comparing bol-mir-172a between method 2 and the new method (*p* = 0.2) (Fig. 3C). Nonetheless, a trend could be observed as bol-mir-172a could not be detected in one of the samples, which decreased the reproducibility of the method (SD = 11.68). Detection and standard deviation for cel-mir-39 (Cq = 16.57 SD = 0.84) and bol-mir-172a (Cq = 32.77 SD = 1.49) from samples extracted with our protocol seemed to follow a trend in which they performed better than when extracted using method 2 (Cq = 16.97 SD = 1.67 and Cq = 34.66 SD = 3.75 respectively). The low reproducibility of the method is best appreciated in supplementary fig. III where qPCR quality control data is illustrated. Due to the lack of statistical significance no further testing (i.e. effect size calculation) was performed for this comparison of methods.

Finally, the importance of adding RNAsecure in the RNA extraction protocol is highlighted in Fig. 3. Samples that were incubated without RNAsecure were significantly worse in detecting spike-ins cel-mir-39 (*p* = 0.04) and UniSp6 (*p* = 0.029) than when RNAsecure incubation was performed. In this case, a strong size effect ratio can also be observed for both the spike-ins (d = 2.0) and bol-mir-172 (d = 6.8). Most likely these results were caused by a partial/total inhibition of the PCR by elements that were not removed during purification such as RNAses, proteins or RNA inhibiting chemicals. Further analysis of the data (Supplementary fig. IV) shows a matching profile in the amplification curves of both spike-ins, reinforcing the PCR inhibition hypothesis. Finally, in none of the samples bol-mir-172a (Cq threshold 35) were detected.

## 4 Discussion

As research continues to show the presence of diet-derived miRNAs in human plasma (Del Pozo-Acebo et al., 2021), it has become a pressing issue to study the fate of miRNAs present in food matrices during digestion. Thus, understanding the potential bioavailability of diet-derived miRNAs is needed, especially in sight of the reported interactions with gene expression (Zhang et al., 2012). Here, gastrointestinal digestion is of particular importance, as we perceive this stage as one of the most critical steps for miRNA stability (Del Pozo-Acebo et al., 2021). Unfortunately, we discovered that frequently utilized methods for RNA isolation, including phenol-chloroform precipitation or spin column (silica based) extraction failed to isolate RNA from the GID or were not consistent in doing so. We hypothesize that this limitation may be the reason why there are studies reporting on diet-derived microRNA survival in oral (Qin et al., 2022) and gastric digestions (Liao et al., 2017; Philip et al., 2015), but not following gastro intestinal digestion.

Therefore, we have optimized a two-step method that combines RNAse inactivation, removal of contaminants from the gastrointestinal matrix and miRNA extraction from the bioaccessible phase of GID with high reproducibility and in less than 2 h. A consensus model of static digestion (Brodkorb et al., 2019) was chosen as a commonly used model of GID.

While working on the method, we identified the main challenges the GID posed for RNA extraction. First, cross-reactivity between the GID and the phenol-chloroform mixture used in the extraction caused a co-precipitation of chemicals from the GID with the RNA in the eluate, as determined by UV–Vis. Moreover, we believe that cross-reactivity with the silica column may have reduced the binding capacity of the latter to RNA. Second, unusually high levels of RNAse activity present in the matrix, similar to as reported earlier in other tissues such as pancreas (Lu et al., 2018), were apparently not inhibited by the methods typically used in extraction (i.e. phenol-chloroform). Based on studies on other RNAse rich tissues, such as pancreas or the placenta (Augereau et al., 2016; Haimov-Kochman et al., 2006), the remaining RNases would then degrade both spike-in controls and miRNAs already present in the GID that would become devoid of any protective elements (e.g. exosomes, proteins) during the purification protocol (Pasloske and Wu, 2004). It is then not uncommon that new strategies adapt to the uniqueness of each specific tissue or fluid to improve the yield of RNA, sometimes reducing RNAse activity, removing potential contaminants or adapting the input volume (Haimov-Kochman et al., 2006; Pagani et al., 2023; Pandit et al., 2013).

It has earlier been shown that both characteristics described above (i.e. complex matrices and high concentration of RNAses) can significantly reduce isolation of RNA. For example, it is well known that glycogen rich samples from the liver cannot easily be processed with standard protocols, as the glycogen usually co-precipitates with the RNA, decreasing the yield and purity (Sharma et al., 2019). Similar effects may be anticipated with starch-rich food items, due to similarities in its structure to glycogen (Wang et al., 2012). Equally, numerous methods have been developed for extracting RNA from other RNAse rich tissues (e.g. pancreas), as normal methods failed to produce high quality RNA (Augereau et al., 2016; Jun et al., 2018). However, to the best of our knowledge, no previous study has investigated and described the impediments for isolating miRNA from GID.

Therefore, here we present an optimized method for miRNA extraction from the bioaccessible phase of the GID. This will provide researchers with a tool to overcome the limitations previously outlined. Furthermore, it enables the study of GID in a standardized, reproducible, and quick (<2h) way. In this case, we overcame the limitations of other methods by adding RNAsecure™ in a 24 times dilution of the stock solution. In addition, we included a kit (miRNeasy Serum/Plasma Advanced) that allowed a high input volume of sample (600 μl) and does not require phenol/chloroform in the extraction to reduce cross-reactivity and achieve a successful extraction of miRNAs from GID matrix.

In addition, we have tested the new method against already existing protocols for miRNA extraction. We have found out that the new protocol was more efficient than other methods available regarding RNA extraction and applicability of downstream reactions (i.e. RT-qPCR) as illustrated by cel-mir-39 and bol-mir-172, being detected earlier by qPCR when the sample was extracted with our method. Furthermore, we have tested whether DNase I incubation prior to extraction may affect the efficiency of the purification. Although our results are non-significant we advise against its use, as it could in some cases reduce the yield, and the DNA is expected to be eliminated successfully in the method as stated by the manufacturer. Unfortunately, further data regarding extraction purity could not be produced, as the low concentrations of RNA in the GID (<10 ng/μl) (Method 1) and carryover of chemicals (Method 2, new method, new minus without RNAsecure) did not allow quantification with traditional methods for RNA (i.e. UV–Vis). Due to the poor results from UV–Vis examinations, and the lack of sensitivity caused by low concentrations of RNA within the sample, these data were not included.

In short, the protocol offers a purification of miRNA from GID based on a combination of an RNAse denaturing buffer (RNAsecure) together with a lysis buffer containing guanidine thiocyanate and detergents, which decrease the cross-reactivity originating from use of phenol/chloroform. Meanwhile, RNAsecure inactivates the RNAses and helps in the denaturing and precipitating other proteins in the sample (Konigsberg, 1972; Pasloske and Wu, 2004). Overall, this leads to a higher recovery of the spike-in cel-mir-39 than other available protocols. We believe that the recovered amounts of diet-derived miRNAs are an accurate representation of the GID contents. The current protocol is a development based on existing techniques, although there is still room for improvement. The RNA extraction from GID still under-performs in terms of efficiency and reproducibility than what is expected for some other tissues and matrices. Even though part of this variability may be caused by the unique media that is the GID, which may not always be a homogeneous dilution, we are aware that an important part of these limitations originates from a technical perspective. Therefore, a challenge for further optimization of the method remains.

As indicated, some limitations apply to the current method. Mainly, it has been developed using cauliflower as the digested plant matrix due to previous reports of bol-mir-172 having a high expression in the *Brassica oleracea* species (of which cauliflower is a cultivar) and its potential survival to GID (Liang et al., 2014). We have speculated that the major cause for reduced yield in miRNA extraction observed with other methods was caused by the components of GID itself, i.e. enzymes and bile acids, and not the food matrix. To fully validate our hypothesis the applicability of the method needs to be extended for other food matrices (both of animal and plant origin). Furthermore, the sample size (*N* = 4) used for testing is limited and higher sample sizes would provide more robust statistics, especially in cases where a trend was observed (i.e. use of DNAse treatment). For example, a higher replicate amount would enable more statistical testing, such as confidence intervals, which were not calculated here due to sample size limitations. Future improvements of the method should consider further reducing cross- reactivity of the GID, as well as adapting the volumes and ratios of wash and binding buffers in a manner that increase the binding conditions to the silica columns and minimize the effect of the GID.

Regardless of the limitations, the development of a novel technique for miRNA extraction in the GID enables the study of diet-derived RNA in biological systems following oral uptake. Among the potential applications of the method are the study of gastrointestinal stability of diet-derived miRNAs, or their response to different digestive conditions. This has been so far a gap in the literature, probably caused by the issues in extraction from gastrointestinal digesta (Philip et al., 2015; Qin et al., 2022). In addition, the protocol will allow to query different diets and food matrices to identify prominent miRNAs that may be associated with these, enabling the use of miRNAs as biomarkers of food intake. Furthermore, availability to easier and more reproducible methods for miRNA extraction, such as the one described here, may boost research into diet-derived miRNAs, facilitating the establishment of these molecules as new food bioactives. It must also be remarked that although the protocol has been developed with a focus on diet-derived miRNAs, this does not exclude the extraction of endogenous miRNAs present within the GID that is also possible. Finally, the insights here gained may benefit the broader miRNA community by providing new approaches for miRNA extraction in complex matrices and tissues.

## Acknowledgements

The authors are grateful for the support of the Luxembourg National Research Fund (PRIDE21/16749720/NEXTIMMUNE2). The technical assistance of Mélanie Vausort and Bernadette Leners is acknowledged.

## CRediT authorship contribution statement

**Miguel Cifuentes Acebal:** Writing – original draft, Methodology, Investigation, Formal analysis, Conceptualization. **Yvan Devaux:** Writing – review & editing, Supervision, Data curation, Conceptualization. **Torsten Bohn:** Writing – review & editing, Supervision, Resources, Methodology, Funding acquisition, Conceptualization.

## Declaration of competing interest

The authors declare the following financial interests/personal relationships which may be considered as potential competing interests: Torsten Bohn reports financial support, administrative support, and travel were provided by Luxembourg Institute of Health. Miguel Cifuentes Acebal reports financial support was provided by National Research Fund Luxembourg. If there are other authors, they declare that they have no known competing financial interests or personal relationships that could have appeared to influence the work reported in this paper.

## Data Availability

No data was used for the research described in the article.

## Appendix A Supplementary data

Step by Step protocol for RNA extraction from GID digesta Supplementary material 1

Additional qPCR data Supplementary material 2
