# Supplementary material for: A protocol for microRNA extraction from gastrointestinal digesta
Source: Food Chem (Oxf). 2025 Feb 11;10:100245. doi: 10.1016/j.fochms.2025.100245 (PMC11889629; doi:10.1016/j.fochms.2025.100245)
Supplement: Step by Step protocol for RNA extraction from GID digesta [file mmc1.docx]

Step by Step protocol for RNA extraction from GID digesta

IMPORTANT: Gastrointestinal Digesta should be kept at -80ºC to prevent further RNA degradation prior to extraction

Note: The protocol is designed so that stops can be done at the end of each part.

Note: Unless otherwise stated all the steps have been conducted at room temperature (20 – 25 ºC).

Note: The centrifuge used for this experiment was a fixed angle centrifuge. The temperature of the chamber was set at 20ºC.

Before starting:

- Thaw the Gastrointestinal digesta (GID) stored at -80^o^C.
- Preheat the heating block at 60ºC

**Part A – RNAse Inhibition/Digestion (15 minutes)**

1. Thoroughly mix the thawed GID by vortexing for a few seconds
2. In a 2 ml micro centrifuge PCR clean tube, add 24 µl RNAsecure ™ to 576 μl GID for a final volume of 600 μl.
3. Mix well by vortexing
4. Incubate the mixture at 60ºC for 10 minutes in a heating block with shaking at 300 rpm (shaking is optional)
5. Remove the sample from the incubator and proceed with Part B

*--- Stop here if needed---*

**Part B – RNA Purification (1 Hour)**

1. Add 180 μl of Buffer RPL to the 600 μl sample.
2. Add 3.5 μl of the spike-in control.
3. Close the tube caps and vortex for 5 s. Incubate for 3 min.
4. Add 60 μl Buffer RPP.
5. Close the tube caps and mix vigorously by vortexing for 20 s. Incubate for 3 min.
6. Centrifuge at 12 000 x g for 3 min to pellet the precipitate. Important: Do not touch the pellet or the walls. Do not aspirate the low-density residue in the top layer. Carryover of these elements may reduce yield
7. Transfer supernatant (~750 μl for 600 μl GID) to a new 2 ml micro centrifuge tube.
8. Add 1 volume of 99.5% isopropanol (750 μl). Mix well by vortexing.
9. Transfer 700 μl of sample to an RNeasy UCP MinElute column. Close the lid, and centrifuge for 15 s at 8 000 x g. Discard the flow-through.
10. Repeat the previous step with the remainder of the sample.
11. Pipet 700 μl Buffer RWT to the RNeasy UCP MinElute spin column. Close the lid, and centrifuge for 15 s at 8 000 x g. Discard the flow-through.
12. Pipet 500 μl Buffer RPE onto the RNeasy UCP MinElute spin column. Close the lid, and centrifuge for 15 s at 8 000 x g. Discard the flow-through.
13. Add 500 μl of 80% ethanol to the RNeasy UCP MinElute spin column. Close the lid, and centrifuge for 2 min at 8 000 x g to wash the spin column membrane. Discard the flow-through.
14. Place the RNeasy UCP MinElute spin column in a new 2 ml collection tube. Open the lid of the spin column and centrifuge at 16 000 x g speed for 5 min to dry the membrane. Discard the flow-through and the collection tube.
15. Place the RNeasy UCP MinElute spin column in a new 1.5 ml collection tube. Add 20 μl RNase-free water directly to the center of the spin column membrane and incubate 1 min.
16. Close the lid, and centrifuge for 1 min at 16 000 x g to elute the RNA.
