# Supplementary material for: A protocol for microRNA extraction from gastrointestinal digesta
Source: Food Chem (Oxf). 2025 Feb 11;10:100245. doi: 10.1016/j.fochms.2025.100245 (PMC11889629; doi:10.1016/j.fochms.2025.100245)
Supplement: Additional qPCR data [file mmc2.docx]

Supplementary Figure I. qPCR data comparing the effect of including a DNA digestion step in the miRNA extraction protocol as opposed to no digestion. Horizontal line indicate statistical significance. A: Results for spike-in cel-mir-39. B: results for bol-mir-172a. data displayed after using cel-mir-39 for normalizing the Cq (∆Cq). N=4.

Supplementary Figure II. Amplification curve, Melt curve and Melt peak from RT-qPCR of samples derived from method 1 (phenol-chloroform with column extraction). A: cel-mir-39 B: UniSp6 C: bol-mir-172a.

Supplementary Figure III. Amplification curve, Melt curve and Melt peak from RT-qPCR of samples derived from method 2 (phenol-chloroform without column extraction). A: cel-mir-39 B: UniSp6 C: bol-mir-172a.

Supplementary Figure IV. Amplification curve, Melt curve and Melt peak from RT-qPCR of samples derived from following the proposed protocol for RNA purification, but excluding the first step 1 (incubation with RNAsecure). A: cel-mir-39 B: UniSp6 C: bol-mir-172a.
